# Antibacterial, Antioxidant, and Antiproliferative Activities of *Corymbia citriodora* and the Essential Oils of Eight *Eucalyptus* Species

**DOI:** 10.3390/medicines5030061

**Published:** 2018-06-21

**Authors:** Maria Graça Miguel, Custódia Gago, Maria Dulce Antunes, Soraia Lagoas, Maria Leonor Faleiro, Cristina Megías, Isabel Cortés-Giraldo, Javier Vioque, Ana Cristina Figueiredo

**Affiliations:** 1 Departamento de Química e Farmácia, Faculdade de Ciências e Tecnologia, Universidade do Algarve, MeditBio, Campus de Gambelas, 8005-139 Faro, Portugal; 2 MeditBio, Faculdade de Ciências e Tecnologia, Universidade do Algarve, Campus de Gambelas, 8005-139 Faro, Portugal; cgago@ualg.pt; 3 CEOT/MeditBio Faculdade de Ciências e Tecnologia, Universidade do Algarve, Campus de Gambelas, 8005-139 Faro, Potugal; mantunes@ualg.pt; 4 Centre for Biomedical Research (CBMR), Faculdade de Ciências e Tecnologia, Universidade do Algarve, Campus de Gambelas, 8005-139 Faro, Potugal; soraia_s_s@hotmail.com (S.L.); mfaleiro@ualg.pt (M.L.F.); 5 Instituto de la Grasa (C.S.I.C.), Universidad Pablo de Olavide, Edificio 46, Carretera de Utrera, km 1, 41013 Sevilla, Spain; cmegias@cica.es (C.M.); icortes@ig.csic.es (I.C.-G.); jvioque@cica.es (J.V.); 6 Centro de Estudos do Ambiente e do Mar (CESAM Lisboa), Faculdade de Ciências da Universidade de Lisboa, Centro de Biotecnologia Vegetal (CBV), DBV, C2, Campo Grande, 1749-016 Lisboa, Portugal; acsf@fc.ul.pt

**Keywords:** *Corymbia citriodora*, *Eucalyptus dives*, *Eucalyptus globulus*, *Eucalyptus delegatensis* subsp. tasmaniensis, *Eucalyptus pauciflora*, *Eucalyptus radiata*, *Eucalyptus smithii*, *Eucalyptus urophylla*, *Eucalyptus viminalis*

## Abstract

**Background:** Essential oils (EOs) have shown antimicrobial, antioxidant, and antiproliferative activity, which may, alone or in combination with other substances, potentially be used for the development of new drugs. However, their chemical variability, depending on the species, varieties, or geographical origin (among other factors) determines different bioactivities that need to be evaluated. **Methods:** The antioxidant activity of *Corymbia citriodora* and eight *Eucalyptus* species EOs was determined using two different methods: the scavenging ability of 2,2′-azino-bis(3-ethylbenzothiazoline-6-sulphonic acid) (ABTS^+•^) and peroxyl free radicals or oxygen radical absorbance capacity (ORAC). Antibacterial activity was evaluated using the microorganisms *Streptococcus pneumoniae* (strains D39 and TIGR4), and *Haemophilus influenza* (strain DSM 9999). The essential oils’ minimum inhibitory concentration (MIC) and minimum bactericidal concentration (MBC) was assessed using a microdilution method. The antiproliferative activity was determined using the THP-1 cell line (human acute monocytic leukaemia) with methylthiazolyldiphenyl-tetrazolium bromide assay (MTT). **Results:** *Corymbia citriodora* and *Eucalyptus viminalis* EOs showed the highest ABTS and peroxyl free radical scavenging capacity. *Eucalyptus globulus* EO showed a high potential to treat *Streptococcus pneumoniae* infections. *Haemophilus influenzae* was the respiratory pathogen that showed the highest resistance to all EOs, including tea tree EO. After 96 h of incubation, at 25 μg/mL, *Eucalyptus radiata* and *Eucalyptus viminalis* EOs showed highest cytotoxic activity against the THP-1 cell line. **Conclusions:** Despite their specific bioactivities, no single EO showed simultaneously good antioxidant, antimicrobial, and antiproliferative activity.

## 1. Introduction

*Streptococcus pneumoniae*, *Staphylococcus aureus*, and *Haemophilus influenzae* may asymptomatically colonize the upper respiratory tract and are the etiologic agents of acute otitis media, sinusitis, and pneumonia. *S. pneumoniae* is also responsible for bacterial meningitis [1,2]. Nowadays, the treatment of these diseases is threatened by the development of antibiotic resistance worldwide [3]. Essential oils (EOs) and their isolated compounds have been shown to possess antimicrobial activity against several pathogens [4]. The complex mixtures that generally constitute EOs can be advantageous because pathogens have difficulty in displaying resistance mechanisms to the multiple constituents of essential oils [5,6].

Reactive oxygen species (ROS) have a physiological role in normal cell function, but when their production exceeds their elimination, an excess of ROS in the body occurs and consequently, diseases also arise because ROS can damage important biomolecules [7]. Damage of nucleic acids may trigger cancer development. In general, it is considered that the use of antioxidants prevents or retards the appearance of diverse diseases, including cancer, although le Gal et al. [8] reported that increased intracellular antioxidant activity may induce tumour cell survival. EOs have been demonstrated to possess antioxidant and antiproliferative properties which may contribute to the development of new chemotherapeutic drugs in the future [9].

The Myrtaceae eucalyptus group comprises four genera: *Angophora* Cav., *Arillastrum* (Brongniart & Gris) Pancher ex Baillon, *Corymbia* K. D. Hill & L.A.S. Johnson, and *Eucalyptus* L’Herit [10,11]. Despite this classification, it is common to designate as *Eucalyptus* several species of the four genera [12]. The eucalyptus group includes around 900 species and subspecies (Brooker and Kleinig in [13]). Species of the genus *Eucalyptus* are well adapted to diverse edaphoclimatic conditions, show rapid growth, and are easy to cultivate. Hence the genus *Eucalyptus*, originally from Australia, is widespread all over the world. *Eucalyptus* is mainly cultivated for obtaining cellulose, wood, gum, and EOs. The latter is obtained from the leaves and used in medicine, perfumery, and the food industry [14].

The recent compilation by Dhakad et al. [15] shows that *Eucalyptus* spp. leaf EOs possess biological and pharmacological properties, namely antimicrobial, antidiabetic, anthelmintic, antiviral, antihistaminic, anti-inflammatory, antimalarial, antioxidant, cytotoxic, larvicidal, nerve blocking, and pain-relieving activity, and are also useful for the relief of respiratory diseases and for wound healing. *Eucalyptus* EOs also possess activities as insecticides, pesticides, and nematicides [15,16,17].

*Eucalyptus* leaf essential oils are usually dominated by monoterpenes and sesquiterpenes. Nevertheless, their relative amounts or the ratio between them may differ according to species and varieties. Even within the same variety, the chemical composition may change depending on the geographical origin [12]. This highlights the importance of the knowledge of chemical composition since it will determine the bioactivity of each EO.

In the present work, the antioxidant, antimicrobial, and antiproliferative activities of EOs from *Corymbia citriodora*, *Eucalyptus dives*, *Eucalyptus globulus*, *Eucalyptus delegatensis* subsp. *tasmaniensis*, *Eucalyptus pauciflora*, *Eucalyptus radiata*, *Eucalyptus smithii*, *Eucalyptus urophylla*, and *Eucalyptus viminalis* (all from Portugal) were evaluated.

## 2. Materials and Methods

### 2.1. Plant Material, EO Extraction, and Composition Analysis

EOs were isolated from fresh aerial parts, collected during the vegetative phase in Portugal (Table 1), and their composition determined as previously described [18].

#### 2.1.1. Isolation of the Essential Oils 

The essential oils were isolated by hydrodistillation for 3 h using a Clevenger-type apparatus according to the European Pharmacopoeia method [19]. The isolation procedure was run at a distillation rate of 3 mL/min, and the essential oils were stored at −20 °C in the dark until analysis.

#### 2.1.2. Gas Chromatography (GC) 

Gas chromatographic analyses were performed using a Perkin Elmer Autosystem XL gas chromatograph (Perkin Elmer, Shelton, CT, USA) equipped with two flame ionization detectors (FIDs), a data handling system, and a vaporizing injector port into which two columns of different polarities were installed: a DB-1 fused-silica column (100% dimethylpolysiloxane, 30 m × 0.25 mm i.d., film thickness 0.25 μm; J & W Scientific Inc., Folsom, CA, USA) and a DB-17HT fused-silica column ((50%-phenyl)-methylpolysiloxane, 30 m × 0.25 mm i.d., film thickness 0.15 μm; J & W Scientific Inc.). The oven temperature was programmed to 45–175 °C, increasing at 3 °C/min, subsequently increased to 300 °C at to 15 °C/min, and then maintained isothermal for 10 min; injector and detector temperatures were 280 °C and 300 °C, respectively. The carrier gas, hydrogen, was adjusted to a linear velocity of 30 cm/s. The samples were injected using a split sampling technique, ratio 1:50. The volume of injection was 0.1 μL of a distilled *n*-pentane-oil solution (1:1). The percentage composition of the oils was computed by the normalization method from the GC peak areas, calculated as a mean value of two injections from each oil, without response factors.

#### 2.1.3. Gas Chromatography-Mass Spectrometry (GC-MS)

The GC-MS unit consisted of a Perkin Elmer Autosystem XL gas chromatograph, equipped with DB-1 fused-silica column (30 m × 0.25 mm i.d., film thickness 0.25 μm; J & W Scientific, Inc.), and interfaced with Perkin-Elmer Turbomass mass spectrometer (software version 5.4.2.1617, PerkinElmer, Shelton, CT, USA). Injector and oven temperatures were as above; transfer line temperature, 280 °C; ion source temperature, 220 °C; carrier gas, helium, adjusted to a linear velocity of 30 cm/s; split ratio, 1:40; ionization energy, 70 eV; scan range, 40–300 u; scan time, 1 s. The identity of the components was assigned by comparison of their retention indices, relative to C_9_-C_17_ *n*-alkane indices, and GC-MS spectra from a laboratory-made library based upon the analyses of reference oils, laboratory-synthesized components, and commercial available standards.

### 2.2. Determination of Antioxidant Activity

Antioxidant activity was determined with 2,2′-azino-bis(3-ethylbenzothiazoline-6-sulphonic acid) (ABTS^+•^) free radical scavenging activity and oxygen radical absorbance capacity (ORAC) methods. For the ABTS method, the preformed radical monocation of ABTS^+•^ was generated by dissolving ABTS in water (7 mM), adding potassium persulfate (2.45 mM, final concentration), and allowing the mixture to stand overnight in the dark at room temperature. Before the assay, the ABTS^+•^ solution was diluted with ethanol to obtain a solution with absorbance of 0.70 at 734 nm [20]. For the assay, 10 μL of sample were added to 1000 μL of ABTS^+•^ solution and left to stand for 6 min, after which the absorbance was read spectrophotometrically at 734 nm. For the control, the volume of sample was replaced by ethanol. The antioxidant activity of each sample was calculated as scavenging effect % (IA%, Inhibitory Activity Percentage) = [(A_0_ − A_1_)/A_0_] × 100, where A_0_ is the absorbance of the control, and A_1_ is the absorbance of the sample. The same procedure was repeated using diverse concentrations of Trolox (6-hydroxy-2,5,7,8-tetramethylchroman-2-carboxylic acid) [21]. The values obtained with the samples were compared with the curve for several Trolox concentrations and the final results given as μmol Trolox equivalent (TE)/g EOs. Tests were carried out in triplicate.

For the ORAC assay, fluorescein (FL) was used as fluorescent probe [22]. Briefly, in each well 150 µL of fluorescein working solution and 25 µL of the previously diluted sample, blank (75 mM phosphate buffer), or standard (Trolox) were added. The plate was covered with a lid and incubated in the preheated (37 °C) BioTek Synergy^TM^ 4 Hybrid Microplate Reader (BioTek, Swindon, UK), country) for 10 min after shaking for 3 min. Then 2,2′-Azobis-2-methyl-propanimidamide dihydrochloride (AAPH) was added to each well of the plate, except for the control and blank wells. The final volume of the assay was 200 µL. The fluorescence was read every minute for 90 min at 485 nm excitation and 527 nm emissions. ORAC values were calculated according to [23]. Briefly, the net area under the curve (AUC) of the standard (Trolox) and samples was calculated. The standard curve was obtained by plotting Trolox concentrations against the average net AUC of the measurements for each concentration. Final ORAC values were calculated using the regression equation between Trolox concentration and the net AUC and were expressed as µmol TE/g EO [24]. Tests were carried out in triplicate.

### 2.3. Determination of Antibacterial Activity

Antibacterial activity was determined against two strains of *Streptococcus pneumoniae* D39 and TIGR4, and *Haemophilus influenza* DSM 9999. Bacterial strains were kept at −70 °C in brain heart infusion (BHI) (Oxoid) supplemented with glycerol (final concentration 25%, *v*/*v*) until use. The cultures were recovered from freezing by growth in brain heart infusion supplemented with 5% (*v*/*v*) horse blood at 37 °C in microaerophilic conditions. Prior to the assay bacterial cultures were grown in Columbia agar (Oxoid) plates supplemented with 5% (*v*/*v*) horse blood at 37 °C in microaerophilic conditions for 24 h. From this plate a suspension with a turbidity value corresponding to 0.5 in the McFarland scale was prepared. From this suspension 0.1 mL was transferred to a new Columbia agar plate supplemented with 5% (*v*/*v*) horse blood. Sterile filter paper discs of 6 mm (Oxoid) were distributed on the agar surface and 3 μL of each essential oil was dispensed in the paper discs. Tea tree essential oil with 38% terpinen-4-ol (Optima Healthcare Ltd., Bradford, UK) was included as reference. Sterile *n*-propanol (essential oil solvent) and the antibiotic chloramphenicol (30 μg/disk) were used as control. Inhibition zones were evaluated after 24 h at 37 °C in microaerophilic conditions. The assays were done in triplicate.

The minimum inhibitory concentration (MIC) for the tested EOs was determined using a microdilution method [6,25]. Concentrations ranging from 0 to 0.4 mg/mL with increments of 0.05 mg/mL were tested. Three replicates for each bacterium were used. The recovery of the viability of each bacterium was evaluated, and the minimum concentration at which no viability was observed was considered as the minimum bactericidal concentration (MBC).

### 2.4. Determination of Antiproliferative Activity

Human leukaemic cell line THP-1 cells were cultured in Dulbecco’s Modified Eagle Medium (DMEM) supplemented with 10% (*v*/*v*) foetal bovine serum, 1% (*v*/*v*) nonessential amino acids, 100U/mL penicillin, and 100 μg/mL streptomycin, according to [24]. They were incubated at 37 °C in a humidified 5% CO_2_ atmosphere.

The growth inhibitory effect of EOs was measured using the 3-(4,5-dimethylthiazol-2-yl) 2,5-diphenyltetrazolium bromide (MTT) assay [26]. THP-1 cells were seeded in 96-well plate at 5 × 10^3^ cells/well and submitted to different EO concentrations (10–500 μg/mL). All samples were dissolved in dimethyl-sulfoxide (DMSO). The solvent concentration in the incubation medium never exceeded 0.5%. Control cultures had the equivalent concentration of DMSO. After treatment, cells were incubated (1 h) in the usual culture conditions after addition of the same volume of medium containing MTT (2 mg/mL). After this period, 150 μL HCl (0.1 M) in isopropanol was added to dissolve the blue formazan crystals formed owing to the reduction of MTT. Absorbance at 570 nm using a background reference wavelength of 630 nm was measured. Antiproliferative activity was expressed as the mean absorbance values for the negative control (DMSO-treated cells) standardized as 100% absorbance (i.e., no growth inhibition) and results were expressed as absorbance (% of control) versus essential oil concentration [24].

### 2.5. Statistical Analysis

Data were analysed by one-way analysis of variance (ANOVA) using IBM SPSS Statistics version 23 (IBM, Armonk, NY, USA). Tukey’s test was used to determine the difference at 5% significance level.

## 3. Results and Discussion

### 3.1. Antioxidant Activity

The antioxidant activity of the EO was determined with an electron reaction-based method (the ABTS+ method) and a hydrogen reaction-based method (the ORAC method). With the TEAC method, Cc EO showed significantly higher antioxidant activity (5.08 µmol TE/g EO) than *Eucalyptus* EOs (Table 2). The lowest antioxidant activity was observed with Eg, Ep, Er and Es EOs, without significant differences among them. The activities ranged from 0.10 in Es to 0.30 μmol TE/g in Eg EO. Cc EO showed the highest scavenging peroxyl radical activity (148.55 µmol TE/g EO) along with Ev EO (135.80 µmol TE/g EO) (Table 2). Eu EO (37.14 µmol TE/g EO) showed the lowest peroxyl radical scavenging activity.

Results for the essential oils of Cc and Es are in agreement with those previously reported by Luis et al. [28] for these species. In Luis et al. [28] study, citronellal (78%) was the main component for *Corymbia citriodora* EO, and 1,8-cineole (82%) dominated in *Eucalyptus smithii* EO.

**Figure 1:**
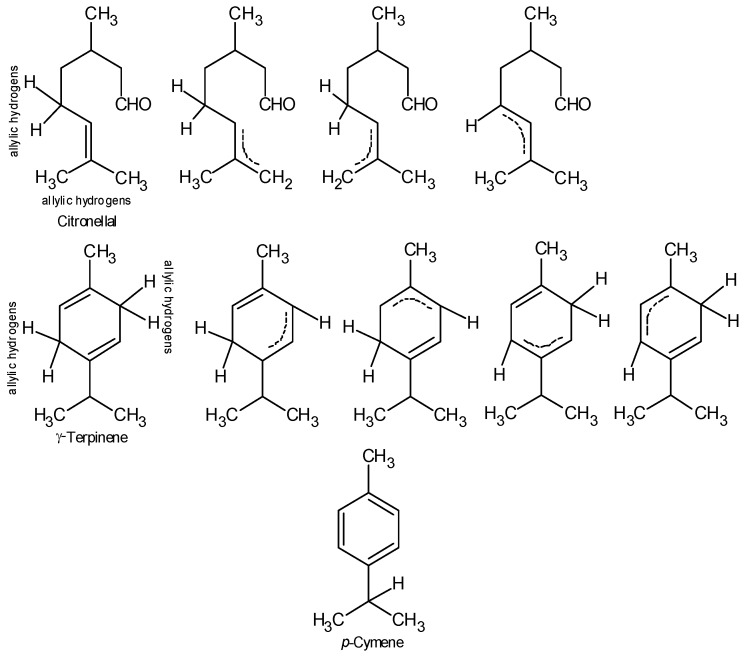
Chemical structure of citronellal, γ-terpinene and *p*-cymene. Citronellal and γ-terpinene representing the allylic hydrogens. The radical formed can be stabilized by resonance.

Citronellal shows moderate antioxidant activity due to its limited capacity to scavenge free radicals [29]. The antioxidant activity of citronellal may be related to one unpaired electron from the molecule allylic hydrogen of both CH_3_ or CH_2_ (Figure 1) that by resonance stabilize the radical formed after the reaction. Nevertheless, Wojtunik et al. [30] considered that only the presence of conjugation of π bonds would allow a resonance-stabilized structure. However, essential oils are complex mixtures and the antioxidant activity cannot be assigned to only one compound. The different constituents of EOs may act synergistically or antagonistically, leading to an overall activity that results from the interaction among several compounds. Ciesla et al. [29] studied the capacity of binary mixtures of some common EO terpenoids, for scavenging DPPH free radicals. They reported an antagonistic effect between citronellal and 1,8-cineole, both present in Cc EO. Nonetheless, the interaction of citronellal with other constituents could trigger a synergistic effect, responsible for the high level of activity observed in Cc EO. Antagonism was also observed by Ciesla et al. [29] for the binary mixture of 1,8-cineole and *p*-cymene. These constituents were the major Er EO compounds (Table 1). Eg, Ep, Er, and Es EOs, which showed the lowest free radical scavenger activities, had 1,8-cineole, α-pinene, and *p*-cymene as major constituents. These monoterpenes were previously reported as poor scavengers of DPPH free radicals [31]. γ-Terpinene, one of the main constituents of Ev EO, is considered a good free radical scavenger [29,32], possibly due to the delocalization of the unpaired electron along the molecule, owing to the two allylic hydrogens that can stabilize the resonance-structures (Figure 1). Moreover, the binary mixture of γ-terpinene and 1,8-cineole (another major constituent of Ev EO) did not produce any significant effects. Despite the presence of an allylic hydrogen in *p*-cymene in conjugation with an aromatic ring, its abstraction from the molecule can be difficult due to steric hindrances, because hydrogen is hidden by the two CH_3_ groups as well as by the aromatic ring (Figure 1).

### 3.2. Antibacterial Activity

Essential oils of thyme, tea tree, peppermint, anise, fennel, and eucalyptus may be used for the treatment of simple respiratory tract diseases [4]. *Eucalyptus* EOs have been used in the treatment of bronchitis, cold, cough, and in the symptomatic relief of catarrh of the upper respiratory tract [4]. Tea tree EO has been used for treating some respiratory infections, such as bronchitis, cold, and influenza [4].

*S. pneumoniae, H. influenzae,* and *Staphylococcus aureus* have been associated with community-acquired pneumonia, along with the less common *Pseudomonas aeruginosa*, *Pneumocystis jirovecii*, *Moraxella catarrhalis*, and other Gram-negative bacteria [33].

In the present work the capacity for inhibiting the growth of two strains of *S. pneumoniae* (D39 and TIGR4) and *H. influenzae* (DSM 9999) by Cc, Ed, Eg, Es and Ev EOs was evaluated and compared with that of tea tree EO and the antibiotic chloramphenicol (Table 3).

All essential oils showed poor antibacterial activity in comparison with the antibiotic chloramphenicol, however differences could be observed among the EOs and bacterial strains (Table 3). For example, all EOs were less effective against *S. pneumoniae* TIGR4 in comparison with *S. pneumoniae* D39. For *S. pneumoniae* TIGR 4, tea tree EO showed better activity than Myrtaceae EOs, and the strain was even resistant to Ed EO. For *H. influenzae*, no significant differences (*p* > 0.05) were observed between the antibacterial activity-assayed Myrtaceae EOs (Table 3).

The MIC and MBC values were determined for Es, Eg, and tea tree EOs (Table 4). The lowest MIC and MBC values were observed for Eg EO against *S. pneumoniae* TIGR 4, with values of 0.015 and 0.05 mg/mL, respectively. Both *S. pneumoniae* strains showed a similar MIC and MBC for the tea tree EO, namely 0.025 and 0.1 mg/mL, respectively. However, for the Es EO *S. pneumoniae* D39 showed a similar MIC value to *S. pneumoniae* TIGR 4, but the MBC value was the double of that of *S. pneumoniae* TIGR 4, showing an MBC of 0.3 mg/mL in contrast to TIGR 4 that showed an MBC of 0.15 mg/mL (Table 4). Such differences may be associated with serotype differences between the strains. Moreover, Eg EO, that in the diffusion agar test showed a very weak activity against *S. pneumoniae* TIGR 4, displayed an efficient activity in the microdilution assay, even in comparison with tea tree EO. These differences may be related to its low diffusion ability. Intriguing was the resistant behaviour of *H. influenza* that was able to growth at the highest EO concentration tested (0.4 mg/mL). Eg EO showed a better activity against *S. pneumoniae*, that can be related with its richness in 1,8-cineole and α-pinene. In addition, 1,8-cineole prevents the growth of other microorganisms, but when in association with other terpenes in an essential oil, such as camphene, α-pinene, globulol and limonene, it was more efficient against *S. aureus*, methicillin-resistant *S. aureus* (MRSA), *Escherichia coli* and *Candida albicans*, and also biofilms of MRSA and *P. aeruginosa* [34].

### 3.3. Antiproliferative Activity

**Figure 2:**
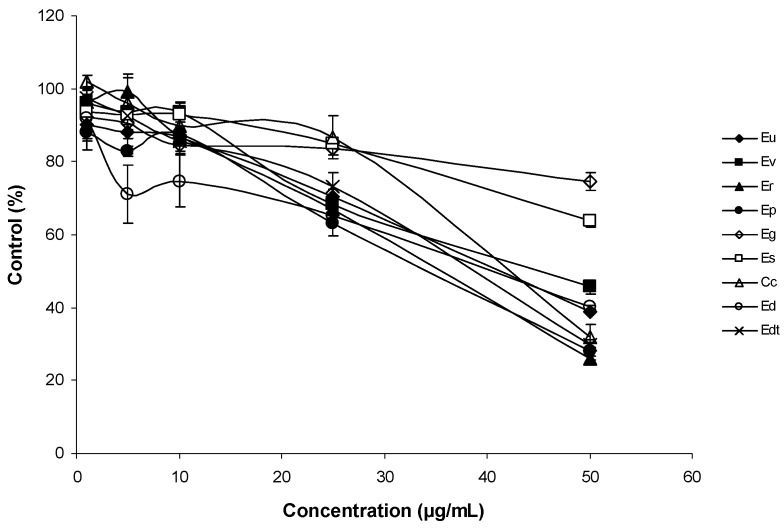
Antiproliferative activity of the EOs on THP-1 cell line after 24 h of exposure. The mean absorbance values for the negative control (dimethyl-sulfoxide (DMSO)-treated cells) were standardized as 100% absorbance (i.e., no growth inhibition) and results were displayed as absorbance (% of control) versus essential oil concentration.

**Figure 3:**
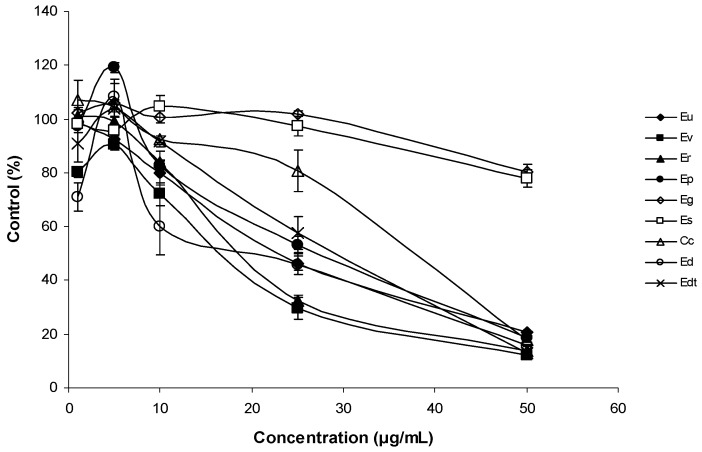
Antiproliferative activity of EOs on THP-1 cell line after 96 h of exposure. The mean absorbance values for the negative control (DMSO-treated cells) were standardized as 100% absorbance (i.e., no growth inhibition) and results were displayed as absorbance (% of control) versus essential oil concentration.

The antiproliferative activities of *Corymbia* and *Eucalyptus* species EOs were studied with the THP-1 cell line (human acute monocytic leukaemia) using the MTT assay. Cells were incubated to increasing amounts of EOs for 24 h and 96 h (Figure 2 and Figure 3).

EOs decreased viability of THP-1 cells in a dose-dependent manner. After one day, a great difference was observed between the antiproliferative activity of Eg and Es EOs and the other EOs, particularly at 50 μg/mL. Hence, Eg and Es EOs possessed the lowest antiproliferative activities. After 96 h incubation of THP1 cells with EOs, the differences were more evident for lower concentrations (10 and 25 μg/mL). At 25 μg/mL, it was possible to distinguish two EOs (Er and Ev) with significantly higher antiproliferative activity than the remaining EOs. At 50 μg/mL, these differences disappeared, because almost all EOs showed survival percentages <20%. The exceptions were Eg and Es EOs, that showed survival percentages above >70%, at 50 μg/mL. The main constituents of Eg EO were 1,8-cineole (64%) and α-pinene (20%), and in Es EO, 1,8-cineole represented 80% of the total EO (Table 1). The major components of the Er and Ev EOs also included 1,8-cineole (48 and 46%, respectively), and other components such as *p*-cymene (13%) for Er and α-pinene (20%), and γ-terpinene (12%) for Ev EOs (Table 1). The results suggested that 1,8-cineole has low antiproliferative activity on the THP-1 leukaemia cell line, although in the presence of *p*-cymene or γ-terpinene, the antiproliferative activity increased.

The weak antiproliferative activity of 1,8-cineole, *p*-cymene, α-pinene has been reported [35], despite these monoterpenes having not been assayed together. Results may suggest a potential synergistic effect between the following pairs: 1,8-cineole and *p*-cymene, and 1,8-cineole with γ-terpinene.

## 4. Conclusions

The highest scavenging of ABTS+ and peroxyl free radicals was observed in Cc and Ev EOs, and the lowest was detected in Eu EO. All essential oils were less effective against *S. pneumoniae* TIGR4 than against the D39 strain. MIC and MBC values showed that Eg EO is promising for the treatment of *S. pneumoniae* infections. EOs displayed low diffusion capacity and the antibacterial activity screening through microdilution test would be more appropriate. *H. influenzae* was the respiratory pathogen that showed the highest resistance to all EOs including that of tea tree. Eg and Es EOs showed the lowest antiproliferative activities on THP-1 cells. After 96 h incubation, the Er and Ev EOs showed the highest antiproliferative activity against THP-1 cells. Although further assays are required, the results suggest that the antiproliferative activity is challenged by the combination of 1,8-cineole with *p*-cymene or with γ-terpinene. Despite their specific bioactivities, none of the assayed EOs showed simultaneously good antibacterial, antioxidant, and antiproliferative capacity.

## Figures and Tables

**Table 1:** Studied *Corymbia* and *Eucalyptus* species. Currently accepted scientific names for plant species, arranged in alphabetic order of the corresponding plant family, sampling year, plant part used for hydrodistillation, plant source, essential oil yield, and main essential oil components (≥10%).

Family/Species		Code	SY	PP	CP	Yield (%, *v*/*w*)	Main Components (≥10%) *
Myrtaceae							
Current accepted species name	Synonyms						
*Corymbia citriodora* (Hook.) K.D.Hill & L.A.S.Johnson ^a^	*Corymbia citriodora* subsp. *variegata* (F.Muell.) A.R.Bean & M.W.McDonald, *Corymbia variegata* (F.Muell.) K.D.Hill & L.A.S.Johnson, *Eucalyptus citriodora* Hook., *Eucalyptus maculata* var. *citriodora* (Hook.) F.M.Bailey, *Eucalyptus melissiodora* Lindl., *Eucalyptus variegata* F.Muell.	Cc	2009	FV	MEE	0.86	citronellal 36, isopulegol 13, citronellol 12, 1,8-cineole 11
*Eucalyptus dives* Schauer ^a^	*Eucalyptus amygdalina* var. *latifolia* H.Deane & Maiden	Ed	2009	FV	MEE	3.30	piperitone 40, α-phellandrene 19, *p*-cymene 19
*Eucalyptus globulus* Labill. ^b^	*Eucalyptus gigantea* Dehnh., *Eucalyptus glauca* A.Cunn. ex DC., *Eucalyptus globulosus* St.-Lag., *Eucalyptus globulus* subsp. *globulus*, *Eucalyptus maidenii* subsp. *globulus* (Labill.) J.B.Kirkp., *Eucalyptus perfoliata* Desf., *Eucalyptus pulverulenta* Link	Eg	2009	FV	Lisbon	2.15	1.8-Cineole 64, α-pinene 20
*Eucalyptus delegatensis* subsp. *tasmaniensis* Boland ^c^	*Eucalyptus gigantea* Hook.f., *Eucalyptus risdonii* var. *elata* Benth., *Eucalyptus tasmanica* Blakely	Edt	2011	FV	MEE	0.52	Limonene 36, *p*-cymene 11, 1,8-cineole 10
*Eucalyptus pauciflora* Sieber ex Spreng ^a^	*Eucalyptus coriacea* A.Cunn. ex Schauer, *Eucalyptus coriacea* var. *alpina* Benth, *Eucalyptus pauciflora* var. *alpina* Ewart, *Eucalyptus pauciflora* subsp. *pauciflora*, *Eucalyptus phlebophylla* F.Muell. ex Miq., *Eucalyptus submultiplinervis* Miq., *Eucalyptus sylvicultrix* F.Muell. ex Benth.	Ep	2009	FV	MEE	0.84	α-pinene 82
*Eucalyptus radiata* A.Cunn. ex DC. ^a^	*Eucalyptus amygdalina* var. *radiata* (A.Cunn. ex DC.) Benth., *Eucalyptus australiana* R.T.Baker & H.G.Sm., *Eucalyptus australiana* var. *latifolia* R.T.Baker & H.G.Sm., *Eucalyptus phellandra* R.T.Baker & H.G.Sm., *Eucalyptus radiata* var. *australiana* (R.T.Baker & H.G.Sm.) Blakel, *Eucalyptus radiata* subsp. *radiata*, *Eucalyptus radiata* var. *subexserta* Blakely	Er	2009	FV	MEE	5.55	1,8-cineole 48, *p*-cymene 13
*Eucalyptus smithii* R.T. Baker ^a^	*Eucalyptus viminalis* var. *pedicellaris* H.Deane & Maiden	Es	2009	FV	MEE	2.80	1,8-cineole 83
*Eucalyptus urophylla* S. T. Blake ^a^	No synonyms recorded	Eu	2009	FV	MEE	0.86	α-phellandrene 45, 1,8-cineole 23
*Eucalyptus viminalis* Labill. ^a^	*Eucalyptus angustifolia* Desf. ex Link, *Eucalyptus gunnii* Miq., *Eucalyptus huberiana* Naudin, *Eucalyptus viminalis* var. *huberiana* (Naudin) N.T.Burb., *Eucalyptus viminalis* var. *rhynchocorys* Maiden, *Eucalyptus viminalis* subsp. *viminalis*	Ev	2009	FV	MEE	1.10	1,8-cineole 46, α-pinene 13, γ-terpinene 12

SY: Sampling year, PP: Plant part, CP: Collection place. * Essential oils previously isolated and chemically characterized in ^a^ Faria et al. [18], ^b^ Barbosa et al. [17] and ^c^ Sena et al. [27] (detailed composition of this EO in Supplementary Table S1). FV–fresh, vegetative-phase aerial parts, MEE: Mata Experimental do Escaroupim arboretum (Salvaterra de Magos, Portugal).

**Table 2:** The activity of essential oils of *Corymbia citriodora* and *Eucalyptus* spp. for scavenging ABTS+ and peroxyl radicals.

Sample Code *	TEAC (µmol TE/g Essential Oil)	ORAC (µmol TE/g Essential Oil)
Cc	5.08 ± 0.08 ^a^	148.55 ± 7.76 ^a^
Ed	0.53 ± 0.08 ^d^	96.83 ± 7.76 ^bc^
Edt	1.27 ± 0.08 ^c^	73.31 ± 7.76 ^c^
Eg	0.30 ± 0.08 ^e^	87.29 ± 7.76 ^bc^
Ep	0.17 ± 0.08 ^e^	99.70 ± 7.76 ^b^
Er	0.21 ± 0.08 ^e^	112.33 ± 7.76 ^b^
Es	0.10 ± 0.08 ^e^	93.66 ± 7.76 ^bc^
Eu	0.68 ± 0.08 ^d^	37.14 ± 7.76 ^d^
Ev	1.62 ± 0.08 ^b^	135.80 ± 7.76 ^a^

* For sample code see Table 1. Values in the same column followed by the same letter are not significant by Tukey’s multiple range test (*p* < 0.05).

**Table 3:** The antibacterial activities of *Corymbia citriodora* and *Eucalyptus* spp. EOs expressed as inhibition zones, in mm. Tea tree oil and chloramphenicol were used as references.

Essential Oil	Microorganism ^†^		
	*S. pneumoniae* D39	*S. pneumoniae* TIGR 4	*H. influenza* DSM 9999
Tea tree	14.00 ± 1.73 ^a^	12.25 ± 1.89 ^a^	11.75 ± 2.36 ^a^
*C. citriodora*	11.33 ± 2.03 ^a^	8.00 ± 1.41 ^b^	11.25 ± 0.50 ^a^
*E. dives*	13.67 ± 2.08 ^a^	NI	10.00 ± 0.81 ^a^
*E. globulus*	12.00 ± 1.73 ^a^	8.75 ± 0.95 ^b^	13.00 ± 1.82 ^a^
*E. smithii*	14.00 ± 1.73 ^a^	8.00 ± 0.81 ^b^	12.00 ± 2.16 ^a^
*E. viminalis*	14.66 ± 1.52 ^a^	6.66 ± 0.57 ^b^	11.75 ± 1.50 ^a^
Chloramphenicol	22.33 ± 4.04 ^b^	21.00 ± 1.15 ^c^	24.00 ± 0.81 ^b^

**^†^** Data represent the mean ± standard deviation of three replicates. Data with the same superscript letter are not significantly different (*p* > 0.05). NI: No inhibition.

**Table 4:** Minimum inhibitory concentration (MIC) and minimum bactericidal concentration (MBC) values for *Eucalyptus* and tea tree EOs ^§^.

Microorganism	Tea Tree		*E. globulus*		*E. smithii*	
	MIC ^†^	MBC ^†^	MIC	MBC	MIC	MBC
*S. pneumoniae* D39	0.025	0.1	0.1	0.15	0.1	0.30
*S. pneumoniae* TIGR 4	0.025	0.1	0.015	0.05	0.1	0.15
*H. influenza* DSM 9999	>0.4	>0.4	>0.4	>0.4	>0.4	>0.4

^§^ Data represent the mean of three independent experiments. **^†^** mg/mL.

## Supplementary Materials

The following are available online at http://www.mdpi.com/2305-6320/5/3/61/s1, Table S1: Percentage composition of the essential oil isolated from *Eucalyptus delegatensis* subsp. *tasmaniensis* Boland, previously included in the study of Sena et al. [27] under the former name of *E. gigantea*.
