# Supplementary material for: Antibacterial, Antioxidant, and Antiproliferative Activities of Corymbia citriodora and the Essential Oils of Eight Eucalyptus Species
Source: Medicines (Basel). 2018 Jun 21;5(3):61. doi: 10.3390/medicines5030061 (PMC6163473; doi:10.3390/medicines5030061)
Supplement: Supplementary file 1 [file medicines-05-00061-s001.pdf]

Supplementary Materials: Antibacterial, Antioxidant, and Antiproliferative Activities of *Corymbia citriodora* and the Essential Oils of Eight *Eucalyptus* Species

Maria Graça Miguel, Custódia Gago, Maria Dulce Antunes, Soraia Lagoas, Maria Leonor Faleiro, Cristina Megías, Isabel Cortés-Giraldo, Javier Vioque and AnaCristina Figueiredo

Table S1. Percentage composition of the essential oil isolated from *Eucalyptus delegatensis* subsp. *tasmaniensis* Boland, previously included in the study of Sena *et al*. [27] under the former name of *E. gigantea*.

Components	RI	*Eucalyptus delegatensis* subsp. *tasmaniensis*
α-Pinene	930	0.9
Camphene	938	t
β-Myrcene	975	t
α-Phellandrene	995	t
α-Terpinene	1002	t
p-Cymene	1003	11.3
1,8-Cineole	1005	9.8
Limonene	1009	36.2
cis-β-Ocimene	1017	0.9
trans-β-Ocimene	1027	t
γ-Terpinene	1035	8.5
cis-Llinalool oxide	1045	t
Fenchone	1050	t
Terpinolene	1064	0.8
Linalool	1074	t
2-Methyl butyric acid isoamyl ester	1074	t
Isopentyl isovalerate	1084	t
allo-Ocimene	1110	t
δ-Terpineol	1134	t
Lavandulol*	1142	1.4
Terpinen-4-ol	1148	2.1
trans-Mentha-1(7),8-dien-2-ol	1159	1.8
α-Terpineol	1159	1.2
trans-Carveol	1189	0.9
cis-*p*-Mentha-1,(7),8-dien-2-ol*	1200	1.2
cis-Carveol	1202	t
Carvone	1210	0.2
Piperitone	1211	0.3
p-Cymen-7-ol	1265	0.6
Thymol	1275	0.5
Carvacrol	1286	0.6
α-Terpenyl acetate	1334	7.4
α-Copaene	1375	t
β-Caryophyllene	1414	2.6
Aromadendrene	1428	t
α-Humulene	1447	0.3
allo-Aromadendrene	1456	0.4
Bicyclogermacrene	1487	0.9
α-Muurolene	1494	t
Calamenene	1505	0.3
δ-Cadinene	1505	0.6
Spathulenol	1551	4.1
β-Caryophyllene oxide	1561	0.6

Viridiflorol	1569	0.4
% Identification		96.8
Grouped components		
Monoterpene hydrocarbons		58.6
Oxygen-containing monoterpenes		28.0
Sesquiterpene hydrocarbons		5.1
Oxygen-containing sesquiterpenes		5.1
Others		t

RI: In-lab calculated retention index relative to C_9-C_{16} *n*-alkanes on the DB-1 column, t: trace (<0.05%).
